# β-Phenylalanine Ester Synthesis from Stable β-Keto Ester Substrate Using Engineered ω-Transaminases

**DOI:** 10.3390/molecules23051211

**Published:** 2018-05-18

**Authors:** Oliver Buß, Moritz Voss, André Delavault, Pascal Gorenflo, Christoph Syldatk, Uwe Bornscheuer, Jens Rudat

**Affiliations:** 1 Institute of Process Engineering in Life Sciences, Section II: Technical Biology, Karlsruhe Institute of Technology, 76021 Karlsruhe, Germany; andre.delavault@kit.edu (A.D.); pascal.gorenflo@kit.edu (P.G.); christoph.syldatk@kit.edu (C.S.); jens.rudat@kit.edu (J.R.); 2 Department of Biotechnology & Enzyme Catalysis, University of Greifswald, Institute of Biochemistry, Felix-Hausdorff-Str. 4, 17487 Greifswald, Germany; moritz.voss@uni-greifswald.de (M.V.); uwe.bornscheuer@uni-greifswald.de (U.B.)

**Keywords:** β-phenylalanine ethyl ester, β-amino acid, ω-transaminase, asymmetric synthesis

## Abstract

The successful synthesis of chiral amines from ketones using ω-transaminases has been shown in many cases in the last two decades. In contrast, the amination of β-keto acids is a special and relatively new challenge, as they decompose easily in aqueous solution. To avoid this, transamination of the more stable β-keto esters would be an interesting alternative. For this reason, ω-transaminases were tested in this study, which enabled the transamination of the β-keto ester substrate ethyl benzoylacetate. Therefore, a ω-transaminase library was screened using a coloring *o*-xylylenediamine assay. The ω-transaminase mutants 3FCR_4M and ATA117 11Rd show great potential for further engineering experiments aiming at the synthesis of chiral (*S*)- and (*R*)-β-phenylalanine esters. This alternative approach resulted in the conversion of 32% and 13% for the (*S*)- and (*R*)-enantiomer, respectively. Furthermore, the (*S*)-β-phenylalanine ethyl ester was isolated by performing a semi-preparative synthesis.

## 1. Introduction

Transaminases (TA) are promising enzymes for the synthesis of chiral amines, amino acids, and amino alcohols. The pyridoxal-5’-phosphate (PLP)-dependent transamination mediates the transfer from an amino donor group onto an acceptor substrate. Aldehydes, ketones, keto acids, or keto esters can serve as the acceptor substrate [1,2]. The reaction mechanism of TA-catalyzed reactions consists of two half-reactions and is described as a ping-pong-bi-bi mechanism [3]. The widespread α-TAs are limited to α-amino acids, containing the amino or keto group in the α position to a carboxylic function. In contrast, ω-TAs are able to transfer amino groups in different positions to the carboxylic moiety or even in the absence of carboxylic functions [4]. 

### Biocatalytic Synthesis of β-Amino Acids

In the last few decades, β-amino acids have gained particular attention as important biological building blocks for the synthesis of artificial peptides and pharmaceuticals [5,6,7,8]. Moreover, β-amino acids show pharmacological effects even as single or cyclized molecules, like β-lactam antibiotics [8,9]. This class of amino acids also gained attention as flavor components [10]. The biocatalytic synthesis of Imagabalin, a chiral β-amino acid containing drug for diabetes treatment, was reported from the β-keto ester precursor, employing an engineered variant of the fold type I (*S*)-selective ω-TA from *Vibrio fluvialis* [1]. To our knowledge, this is the only example of the conversion of β-keto esters using ω-TAs as catalysts. 

A prominent example of β-amino acids in pharmaceuticals is β-phenylalanine (**1**), or rather α-hydroxy-β-phenylalanine, present in the natural substance paclitaxel (Taxol), which is a prominent anti-cancer agent [11]. Moreover, esters of **1** are interesting for the synthesis of nitrogen-containing heterocyclic compounds, e.g., for the synthesis of β-lactams, aminophenylpropanoic acid-terminated polyoxyethylene esters, nicotinamide derivatives for the treatment of respiratory and allergic diseases, (*S*)-dapoxetine (reaction with LiAlH_4_), and for the synthesis of putative anti-amnesiant agents [12,13,14,15,16,17].

Until now, different ω-TA-based methods for the synthesis of optically pure **1**, like chiral resolution or enzyme cascade reactions, have been reported [18,19,20,21]. Thereby, the synthesis from the stable asymmetric β-keto ester is favored for the chiral synthesis of **1**, since the corresponding β-keto acid (benzoylacetic acid) is not stable in water and undergoes decarboxylation. An approach to circumvent the decarboxylation is the creation of a reaction cascade starting from the β-keto ester, using a lipase for the hydrolysis of the ester and ω-TA for the direct conversion of the unstable β-keto acid intermediate [21]. Mathew et al. optimized this cascade and increased the yield of **1** to 44% by employing high concentrations of the *Candida rugosa* lipase (CRL, 20 mg mL^−1^ enzyme) in combination with the ω-TA from *Sphaerobacter thermophilus* [22,23,24]. An alternative cascade also reported by Mathew et al. starts from the corresponding β-keto nitrile, using the β-keto nitrilase from *Bradyrhizobium japonicum* in high concentrations (25 mg mL^−1^ of cell dry weight) to form the unstable β-keto acid intermediate, which is also converted by ω-TA and resulted in 72% yield [22,24]. In unpublished experiments from our group, the lipase-based cascade resulted in only 7.2–16% yield of **1** by applying high concentrations of CRL (20 mg mL^−1^ enzyme) and using 1.2 mg mL^−1^ of purified ω-TA from *Variovorax paradoxus*, known for its high activity towards **1** [25]. However, lower concentrations of CRL (1 mg mL^−1^) resulted in no product formation.

**Scheme 1:**
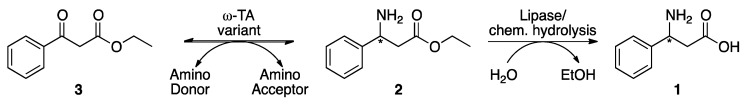
Enzymatic asymmetric synthesis using a β-keto ester as stable substrate alternative in contrast to unstable β-keto acids. Starting with ethyl benzoylacetate (**3**) in combination with an adequate amino donor and ω-TA, the resulting β-phenylalanine ethyl ester (**2**) can be isolated or hydrolyzed to β-phenylalanine (**1**).

To avoid critical cascade intermediates, the asymmetric transamination of the stable aromatic β-keto ester (**3**) would be of advantage and results in the chiral β-phenylalanine ethyl ester (**2**), which can be chemically or enzymatically hydrolyzed to (*R*)- or (*S*)-β-phenylalanine (**1**) (Scheme 1). The obstacle to this efficient biocatalytic approach is the lack of known ω-TAs with activity towards β-keto esters. An exception is the engineered ω-TA variant from *V. fluvialis* with activity towards the aliphatic β-keto ester Imagabalin precursor [1].

On this basis, either an existing ω-TA can be engineered or a screening with a ω-TA library can be performed. Therefore, to enable the proposed direct synthesis of **1** by amination of the stable β-keto ester (**3**), we performed a screening of a ω-TA library to identify variants converting **3** and analyzed the hits with respect to yield and optical purity.

## 2. Results

### 2.1. Identification of Promising ω-TA Variants

The library contained ω-TAs from fold type I and IV of PLP-dependent enzymes to identify variants with (*R*)- and (*S*)-selectivity. Several wild-type transaminases were analyzed as well as engineered variants from *Silicibacter pomeroyi* (PDB ID: 3HMU), *Rhodobacter sphaeroides* (PDB ID: 3I5T), *Ruegeria* sp. TM1040 (PDB ID: 3FCR), and *V. fluvialis* (PDB ID: 4E3Q). Also, the engineered ω-TA from Codexis (ATA117 11Rd; PDB ID: 3WWJ) was included in the screening. The screening was performed with the colorimetric *o*-xylylenediamine (**4**, *o*-xyl) as amino donor, because **4** showed promising acceptance by several commercially available ω-TAs. Additionally, **4** shifts the equilibrium towards the product side due to the polymerization of the formed isoindole co-product [4,26]. The formed polymer results in a clearly visible precipitate, indicating conversion in the screening [26]. The *o*-xyl screening-assay was chosen because other screening methods either use the ketone as a signaling molecule or were not compatible with the selected reaction conditions [27,28,29,30].

**Figure 1:**
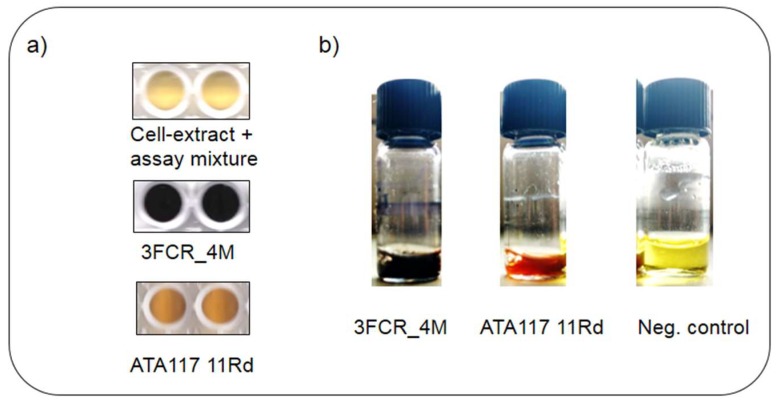
Screening for **3**-converting ω-TAs. (**a**) **4** was used as the amine donor in the screening. High activity resulted in dark precipitates. A yellow color indicates that no or low activity is present. For brown and orange colors it remains unclear whether enzyme activity is present. Cell extracts mixed with assay ingredients are displayed as negative control. (**b**) Result of 1 mL scale experiments after 24 h at 30 °C.

The screening was performed in 96-well MTP format, enabling the simultaneous analysis of various ω-TA variants. After starting the reaction by adding ω-TA lysate, strong staining was observed within the first hours for variants from *Ruegeria* sp. TM1040 ω-TA (3FCR). The staining was clearly visible and resulted in dark precipitation after a 24-h incubation. In addition, the engineered fold type IV ω-TA ATA117 11Rd from Codexis showed a slightly brown staining (Figure 1). Beside those two variants, the ω-TA from *Silicibacter pomeroyi* (3HMU) also showed a marginal color change. However, it is known that only strong changes and precipitation are indicators of substrate conversion [26,31].

The 3FCR_4M mutant and the ATA117 11Rd were chosen as promising candidates for the synthesis of (*S*)- and (*R*)-**2**, respectively. Furthermore, the 3FCR_4M_59L mutant was tested, which was also active but showed no improvement in comparison to 3FCR_4M and was therefore excluded from further experiments.

### 2.2. Asymmetric Synthesis of the β-Phenylalanine Ethyl Ester

For the reproduction and detailed analysis of the activities of the beneficial mutants towards the β-keto ester (**3**), the two ω-TA variants 3FCR_4M and ATA117 11Rd were expressed and purified. Additional to *o*-xyl (**4**), the asymmetric synthesis reactions were carried out with the common amino donors (*R*/*S*)-1-phenylethylamine (**7a**), d/l-alanine, and isopropylamine. These amino donors have a different effect on the equilibrium of the reaction. Since the corresponding keto product of **4** polymerizes and precipitates in the reaction, the equilibrium is naturally shifted towards the side of the desired amine product [26]. The well-accepted amino donor **7a** also shifts the reaction towards the amine products because the corresponding acetophenone inhibits the reverse reaction by conjugation of the aromatic ring with the ketone position [32]. In contrast to these positive side effects, other amino donors like d/l-alanine and isopropylamine are required in high stoichiometric excess to shift the reaction on the product side. A further shift in chemical equilibrium can be achieved by the removal of the corresponding keto product from the reaction. Whereas the volatile acetone (keto product of isopropylamine) can be removed by evaporation, pyruvate (keto product of alanine) is reduced to lactate in a subsequent reaction using the NADH-dependent lactate dehydrogenase (LDH). For efficient pyruvate reduction, the resulting NAD^+^ is recycled by the glucose dehydrogenase (GDH) with an excess of d-glucose, establishing the GDH-LDH cascade system [33].

Isopropylamine had a negative effect on the ester stability in high concentration and was therefore excluded as an amino donor (Figure S2). 

Therefore, **7a**, alanine and **4** were investigated and the DMSO concentration was adjusted to 30% (*v*/*v*) to increase solubility of **1** (Table 1). It turned out that, despite the use of a pyruvate removal system, the equilibrium could not be decisively shifted to the side of the products.

The (*S*)-selective 3FCR_4M variant showed a conversion of 6%, the (*R*)-selective ATA117 11Rd no turnover by using alanine as an amino donor. **7a** also did not prove to be an amino donor with suitable properties for shifting the reaction equilibrium, with conversions below 1% for both transaminase variants. The largest turnover rates were achieved with *o*-xyl (**4**) (32% and 13%), which was previously also used as amino donor for screening. Overall, it was shown that the reactions showed a high enantiomeric purity of 99% ee. Only ATA117 11Rd showed slightly lower enantioselectivity. It was therefore possible to produce both (*S*)- and (*R*)-enantiomers of **1**. Since only the free amino acid could be detected by HPLC, an additional TLC-MS analysis was carried out, which showed that **2** could be produced (Figure S1). In addition to the TLC detection, the β-amino ester was synthesized in a semi-preparative approach with **4** and analyzed by NMR. 

### 2.3. Optimization of the Reaction Condition and Larger Scale Synthesis of **2**

One obstacle for the synthesis on a larger scale was the poor solubility of **3**, even in aqueous solutions with a DMSO content of 30%. The water solubility of the **3** is only 2 mM in pure aqueous solutions [34]. In addition, **3** appeared to be a relatively hydrolysis-sensitive ester, where the reduction of water activity (a_w_) was also a desirable goal. This is due to the fact that hydrolysis leads directly to the formation of the unstable β-keto acid intermediate, which ultimately decarboxylates and thus cannot be converted to either **2** or **1**. The a_w_-value of the DMSO-HEPES solution was determined to be 0.966 (30% *v*/*v*), 0.980 (20% *v*/*v*) and 0.998 for pure HEPES buffer. The pH 7.5 in the reaction mixture is a compromise between ester stability and ω-TA activity. Most ω-TAs perform best at slightly alkaline conditions between pH 7.5 and 9. In contrast, the stability of the ester substrate is higher under slightly acidic conditions. Therefore, a Design of Experiments was carried out and investigated whether higher yields could be achieved by varying the reaction conditions. However, the yield of **2** could not be increased (Tables S2 and S3). ATA117 11Rd was therefore not used for a 200 mL (30 mM) approach, since the yield of **2** was generally many times lower than for 3FCR_4M. The reaction was followed by TLC measurements and after no increase in product concentration was observed, the synthesis was stopped after 48 h. **2** was subsequently purified using flash chromatography. HPLC analysis showed that the enantiomeric purity was above 99% ee.

## 3. Discussion

Up to now, no β-phenylalanine ester product purification has been published for a ω-TA-catalyzed reaction. The total isolated yield of **2** was only 104 mg, representing 9% of the theoretically possible yield, which may be due to the extraction procedure by column chromatography, but also perhaps to the amine ester being unstable and forming free β-phenylalanine. Besides hydrolysis and purification of **2**, another reason for the supposedly low yield could be that ω-TA was not optimized for the conversion of β-keto esters. The substrate concentration of *o*-xyl had little effect on the total conversion rate, suggesting that the co-products inhibit the enzyme or higher concentrations of *o*-xyl have a negative influence on the enzymatic activity. The concentration of alanine as well as the **7a** was already described in other publications as effective [35,36]. However, the 3FCR_4M mutant does not show high activity towards **7a** and the activity of the 3FCR wild type is even lower [35]. It remains questionable whether the molar ratio should be further increased for those substrates in order to obtain higher yields.

The 3FCR_4M variant was engineered by Pavlidis et al. towards the conversion of bulky chiral amines and afterwards applied for the conversion of bicyclic amines by Weiß et al. [35,36]. In contrast, the aminotransferase ATA117 11Rd, harboring 27 mutations, was engineered by Savile et al. from a homolog of an enzyme from *Arthrobacter* sp. KNK168 towards the conversion of the bulky substrate prositagliptin, which is a fluorinated substrate with a large difference in polarity compared to **3** [37]. Furthermore, ATA117 11Rd—the (*R*)-selective fold type IV PLP-dependent enzyme—showed that the synthesis of (*R*)-**2** is possible, albeit at a low turnover rate. However, the general affinity of ATA117 11Rd is relatively low towards *o-*xyl, as shown by Green et al. [26].

One challenge is to find the amino acid residues of particular importance for the acceptance of β-keto esters. Therefore, some amino acid residues within fold type I ω-TA can be noted as putatively important for β-keto ester activity. The ω-TA-catalyzed synthesis of aliphatic β-amino esters has already been shown with an engineered enzyme from *V. fluvialis*. The engineered ω-TA showed one elementary important mutation, W57F, which was crucial for the conversion of the ester substrate ethyl 3-aminohexanoate. The sites of engineering can be easily transferred using the standard numbering scheme of the ω-transaminase engineering database (oTAED) [1,38]. This residue can be relocated within the engineered 3FCR_4M at residue Y59. 3FCR_4M contains, besides the mentioned Y59F mutant, the amino acid exchanges Y87F and Y152F. These amino acid exchanges are beneficial for binding the cofactor–substrate intermediate and towards the expansion of the small binding pocket (also known as the P-pocket). These results clearly demonstrated that the Y59 residue is important for the conversion of β-keto ester substrates and therefore confirming the results of Midelfort et al. In addition, it might be beneficial to mutate the flipping arginine, which is known for dual-substrate recognition in 3FCR, towards phenylalanine, as shown for the *V. fluvialis* engineering. This mutation alone increased the activity of the *V. fluvialis* ω-TA towards the aliphatic β-keto ester substrate about 20-fold.

In general, TAs have never been engineered towards aromatic β-keto ester, so this work should be considered as proof of concept. The synthesis of β-keto acids or esters can be conducted using prochiral substrates and the engineered 3FCR_4M and ATA117 11Rd. Since these enzymes have not been particularly designed for the conversion of ester substrates, further engineering could increase yields and avoid expensive amino donors, like *o*-xyl or laborious equilibrium displacement techniques (e.g. the LDH-GDH cascade system).

## 4. Materials and Methods 

All chemicals, unless indicated otherwise, were purchased from Sigma-Aldrich (St. Louis, Missouri, USA) or Carl Roth (Karlsruhe, Germany) at the highest purity grade (>99%).

### 4.1. Protein Expression and Purification

The genes encoding all ω-TAs for large-scale expression were included in pET vector systems (see Table S1). The enzymes were expressed in the *Escherichia coli* (*E. coli*) BL21 (DE3) strain using auto-induction LB-broth medium with trace elements (Formedium, Norfolk, UK) and purified using Ni-affinity chromatography according to Buß et al. [39].

### 4.2. Screening of the ω-TA Library

For screening the ω-TA library *E. coli* BL21 (DE3) strains were used containing pET or pGASTON plasmids with resistance genes against kanamycin or ampicillin. The concentration of kanamycin was 50 µg mL^−1^ and for ampicillin 100 µg mL^−1^ in the medium for growth and expression. The cells were precultured in 0.2 mL LB medium overnight at 30 °C, 700 rpm in 96-microtiter plates (MTP). From this preculture 20 µL were transferred to inoculate 96-well plates filled with 960 µL TB medium and appropriate antibiotics. The plates were sealed with oxygen permeable membranes (Rotilabo-cling film, Carl Roth). The cells were incubated at 37 °C for 6 h at 700 rpm and the expression was induced by the addition of 0.5 mM IPTG or 0.2% l-rhamnose. The cultures were incubated at 26 °C for 20 h at 700 rpm. For harvesting, the cells were settled at 5000 g for 20 min at 4 °C in a robotic centrifuge (Rotanta 460-Robotic, Hettich GmbH &Co.KG, Tuttlingen, Germany) [40]. The cell pellets were resuspended in 50 mM HEPES buffer pH 7.5 with 0.1 mM PLP for washing the pellets. After centrifuging the pellets (5000 g for 20 min at 4 °C), they were resuspended for lysis in 50 mM HEPES buffer pH 7.5 with 0.1 mM PLP and 1 mg mL^−1^ lysozyme and incubated at 30 °C for 90 min at 700 rpm and the cell debris were sedimented (5000 g for 20 min at 4 °C). The supernatant was transferred in a clean 96-well plate. Fifty microliters of cell-free supernatant was transferred to 150 µL reaction mixture. The final mixture contained 50 mM HEPES buffer pH 7.5, 5 mM of **4**, 7.5 mM of **3** (>95% purity, Sigma-Aldrich), 1 mM PLP, and 10% (*v*/*v*) dimethyl sulfoxide (DMSO). The reactions were performed in 96-well MTPs at 30 °C and 120 rpm overnight. The analyzed ω-TA variants are summarized in Table S1, including the 29 different ω-TAs. 

### 4.3. TLC-(MS) Analysis

For thin-layer chromatography (TLC), 100 µL samples were taken from the enzymatic synthesis mixture and extracted with 200 µL ethyl acetate (EtOAc). The extraction was performed at a shaking rate of 2000 rpm for 5 min in a ThermoMixer C (Eppendorf, Hamburg, Germany) and phases were separated by centrifugation at 13,000 rpm in a benchtop centrifuge (Eppendorf). To analyze the synthesis mixture, a mobile phase consisting of 45% acetone, 25% *tert*-butyl ether, 20% acetic acid, and 10% water was used. For observation of reaction progress, samples of 10 µL were spotted on standard silica TLC plates (Sigma-Aldrich) and stained with 0.5% ninhydrin solution. For determination of product mass, a synthesis sample was spotted 15 times on a TLC plate, separated and analyzed using a thin-layer chromatography mass spectrometer with electron spray ionization (expression CMS, Advion, Ithaca, New York, USA). Samples were taken directly from the TLC plate using an Express-TLC-plate reader and methanol for solubilization. The samples were compared to β-phenylalanine methyl ester (**5**) as reference substance. For blanking the MS signal a TLC spot without substrate/product was taken and subtracted from sample measurements.

### 4.4. HPLC Analysis

Samples from enzymatic synthesis were analyzed using HPLC (Agilent 1200 Series, Santa Clara, California, USA). According to the protocol from Brucher et al., the samples were derivatized with *o*-phthalaldehyde and separated using reversed phase C18 column (150 × 4.6 mm HyperClone 5 µm, Phenomenex Inc.,Aschaffenburg, Germany), which was operated with a gradient of 50 to 60% methanol. As the aqueous solvent, 40 mM sodium phosphate solution (pH 6.5) was utilized [41]. The flow rate started at 0.7 mL min^−1^, decreased to 0.5 mL min^−1^ (after 2 min), and finally increased back to 0.7 mL min^−1^. This allowed for better separation of small amine molecules, which normally elute very fast. The temperature of the column oven was set to 40 °C. The eluted compounds were analyzed with a DAD detector at 337 nm. A calibration was performed using defined concentrations of *rac*-**1**. **1** was diluted in 50 mM HEPES buffer (pH 7.5) and prepared according to the synthesis samples (see small-scale enzymatic synthesis). Optically pure standards from Peptech (Burlington, VT, USA) were used to determine the absolute configuration of the (*R*)- and (*S*)-enantiomers. The retention time of the (*R*)-enantiomer was 3.12 min and that of the (*S*)-enantiomer 3.61 min. 

### 4.5. Small Scale Enzymatic Synthesis 

Enzymatic synthesis of **2** was performed on a 1 mL scale consisting of 50 mM HEPES buffer (pH 7.5), 10 to 30% (*v*/*v*) DMSO, 1 mM PLP, 10 mM **3**, and different amounts of different amino donors. The concentration of the amino donors is given in the results section. In general, 0.2 mg mL^−1^ purified ω-TA was used in the reaction mixture. The LDH-GDH cascade system was prepared according to Weiß et al. [36]. The temperature was set to 30 °C at a 500 rpm shaking rate. Seventy-five microliters from the samples were taken at different time points and the reactions were directly stopped using heat treatment (99 °C), 1 M NaOH, and subsequently neutralized with 1 M HCl for further analysis using HPLC. A second sample was taken and extracted using 100 µL EtOAc for the isolation of **2**. For extraction, the samples were shaken at 2000 rpm for 5 min and analyzed using TLC-(MS).

### 4.6. Design of Experiments

For investigation of the influence of solvent content of DMSO in the reaction medium and for optimization of substrate concentration, a design of experiments was performed using Design Expert 8 with 11 experiments for each selected ω-TA. Three center points were chosen for the experiment setup. Additionally, the concentration of **3** was varied from 10 to 50 mM, **4** from 10 to 100 mM and the content of DMSO (*v*/*v*) was tested in the range from 10 to 30% (*v*/*v*). The response was defined as yield of **2** using GC analysis.

### 4.7. GC Analysis

GC analysis was performed to monitor the concentration of **3**. Therefore, the standard of **2** (Sigma-Aldrich) was first dissolved in 50 mM HEPES buffer pH 7.5 and a dilution series was mixed. The samples were extracted with 1:1 (*v*/*v*) of EtOAc. The two-phases were mixed according to the extraction for TLC experiments. GC-analysis was performed using a 6850N Network GC system from Agilent employing a DB-wax column (30 m (length), 0.25 mm (diameter), 0.25 µm (film)) and flame ionization detection. Two microliters of the sample were injected (split ratio 10:1) at an injector temperature of 250 °C. The separation was achieved using an oven temperature gradient of 40 to 250 °C (8 C min^−1^). The flow rate was set to 0.6 mL min^−1^. **2** was detected at a retention time of 28.9 min. After a retention time of 29.3 min, **3** was detected.

### 4.8. Determination of Water Activity

Water activity (a_w_) was tested using an AquaLab 4TE device (METER Group, Inc., Pullman, WA, USA) with a chilled mirror dew point sensor. The measurements were performed at RT and buffer solutions with different DMSO contents were compared.

### 4.9. Synthesis Scale-up of (S)-phenylalanine Ethyl Ester and Product Purification

For production on a larger scale (200 mL), 30 mM **4** (0.82 g) and 30 mM **3** (1.15 g) were solved in an aqueous 30% (*v*/*v*) DMSO solution. The pH was adjusted to 7.5 with a 100 mM HEPES buffer and 1 mM PLP was added. The reaction was started by adding 20 mg of purified 3FCR_4M. The reaction process was monitored by TLC (solvent: 40% n-hexane, 40% EtOAc, 10% acetic acid, 10% MeOH). The pH value was monitored by pH measuring strips. The reaction was performed in a 500-mL round-bottomed flask at a rotary evaporator at 150 rpm and 30 °C under standard pressure. After 48 h the reaction was stopped by EtOAc extraction with 3 × 150 mL solvent. The solvent was filtrated to remove the dark precipitate and the filtrate was dried using anhydrous MgSO_4_. The extraction solvent was subsequently evaporated at the rotary evaporator and the product was solved in 5 mL EtOAc containing 5% MeOH and 10% acetic acid. The purification was performed using MPLC (Reveleris Prep., BÜCHI Labortechnik AG, Flawil, Switzerland). For separation, a Reverleris PureFlash 4g column and a flow rate of 15 mL min^−1^ was used. Liquid injection was selected and a gradient of *n*-hexane and EtOAc (5% MeOH and 10% acetic acid) was used as follows: 50% for 3.1 min, 50 to 100% EtOAc in 3.1 min, and 100% holding for 3.1 min. To elute **2** a gradient starting with EtOAc and MeOH was performed starting with 95% of EtOAc. Within 6.3 min the concentration of methanol increased to 100%. Peaks were observed using an evaporative light scattering detector and fractions collected. The fractions containing **2** were again evaporated and freeze-dried to remove acetic acid. The product was analyzed using TLC, HPLC (optical purity), and NMR.

### 4.10. Analytical NMR Data of (S)-Phenylalanine Ethyl Ester

*(S)-ethyl 3-amino-3-phenylpropanoate* (**2**) acetate salt as a brown solution. ^1^H NMR (Methanol-*d*_4_, 300 MHz) δ(ppm): 1.17 (3H, t, ^3^*J* = 9 Hz, CH_2_C*H*_3_), 4.57 (1H, dd, ^3^*J* = 9 Hz, ^2^*J* = 15 Hz, CHC*H*_2_CO), 4.72 (1H, dd, ^3^*J* = 6 Hz, ^2^*J* = 15 Hz, CHC*H*_2_CO), 5.68 (2H, q, ^3^*J* = 9 Hz, C*H*_2_CH_3_), 6.27 (1H, t, ^3^*J* = 6 Hz, C*H*NH_2_), 8.97–9.06 (5H, m, C_6_*H*_5_), 9.53 (2H, br s, N*H*_2_). ^13^C NMR (Methanol-*d*_4_, 300 MHz) δ(ppm): 14.36 (CH_2_**C**H_3_), 39.61 (CH**C**H_2_CO), 52.94 (**C**HNH_2_), 62.24 (**C**H_2_CH_3_), 128.42, 130.25, 130.40, 130.86 (**C**_6_H_5_), 171.23 (**C**O).

## Figures and Tables

**Table 1:** Summary of the asymmetric synthesis reactions using different amino donors with respect to yield and optical purity. The conversion refers to **1** after alkaline hydrolysis of the transamination product **2**. The reaction conditions were: 10 mM **3**, variable concentrations of the corresponding amino donors, 30% DMSO and 1 mM PLP in 50 mM HEPES buffer pH 7.5.

Amino Donor			3FCR_4M		ATA117 11Rd	
Amine	Concentration	Eq.	Conversion ^2^	% ee (*S*)	Conversion ^2^	% ee (*R*)
**7a**	50 mM	5	1%	99	<0.5%	n.d.
Alanine ^1^	250 mM	25	6%	99	n.d.	n.d.
**4**	10 mM	1	32%	99	13%	92

^1^l-alanine for 3FCR_4M and d/l-alanine for ATA117 11Rd. The equilibrium was shifted towards the product side by applying the GDH-LDH system [33]. ^2^ The conversion was determined after 24 h. eq. = equivalents of amino donor to amino acceptor. n.d. = no detectable conversion.

## Supplementary Materials

The following Supplementary Materials are available online. Figure S1: TLC-MS analysis of **2**. **2** was synthesized using 3FCR_4M, Figure S2: Pre-test of different amino donors **7a**, isopropylamine and alanine, Figure S3: Gas chromatography for detection of β-phenylalanine ethyl ester, Figure S4: Design of Experiments varying DMSO, *o*-xylylenediamine and β-keto ester concentrations, Figure S5: Reaction batch of **2** on a 200 mL scale, Figure S6: ^1^H-NMR-spectrum of the resulted product after purification using flash chromatography, Figure S7: ^13^C-NMR-spectrum of the resulted product after purification using flash chromatography, Table S1: ω-TA plasmid sources for β-phenylalanine ethyl ester screening, Table S2: Design of Experiments for optimization of β-phenylalanine ethyl ester production using 3FCR_4M, Table S3: Design of Experiments for optimization of β-phenylalanine ethyl ester production using ATA117 11 Rd.
