# Supplementary material for: β-Phenylalanine Ester Synthesis from Stable β-Keto Ester Substrate Using Engineered ω-Transaminases
Source: Molecules. 2018 May 18;23(5):1211. doi: 10.3390/molecules23051211 (PMC6100204; doi:10.3390/molecules23051211)
Supplement: Supplementary file 1 [file molecules-23-01211-s001.pdf]

Supplementary material

β-phenylalanine ester synthesis from stable β-keto ester substrate using engineered ω-transaminases

Oliver Buß*[1], Moritz Voß*[2], André Delavault[1], Pascal Gorenflo[1], Christoph Syldatk[1], Uwe Bornscheuer[2] and Jens Rudat[1]

[1]Institute of Process Engineering in Life Sciences, Section II: Technical Biology, Karlsruhe Institute of Technology, Karlsruhe, Germany
[2]Dept. of Biotechnology & Enzyme Catalysis, University of Greifswald, Institute of Biochemistry, Felix-Hausdorff-Str. 4, 17487 Greifswald, Germany

Corresponding author: oliver.buss@kit.edu

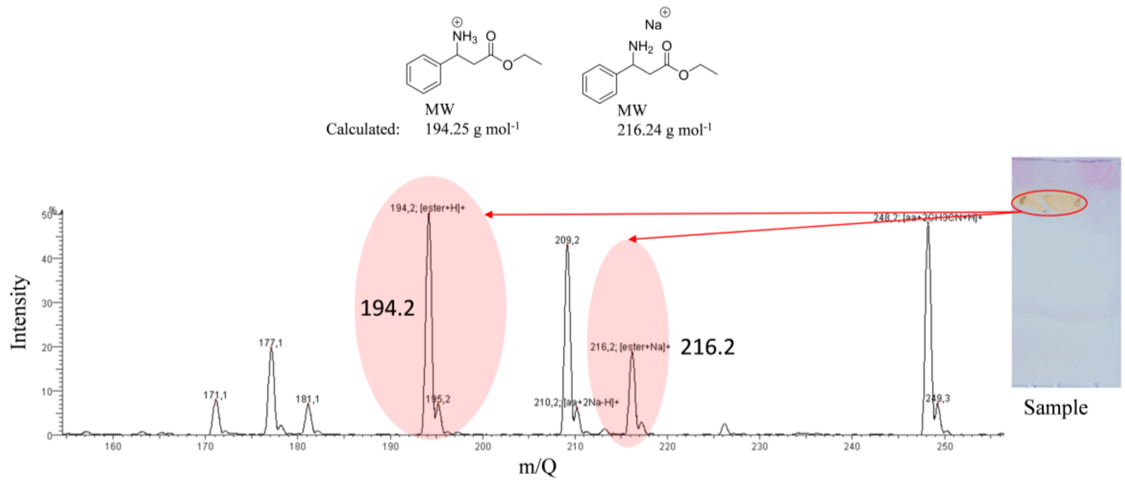

Figure S1 TLC-MS analysis of **2**. **2** was synthesized using the transaminase 3FCR_4M. The resulting MS signals 194.2 (plus proton) and 216.2 (plus sodium) were identified as expected molecular weight (MW) signals for **2**. TLC-MS measurements were verified using methyl-3-amino-phenylpropanoate as standard.

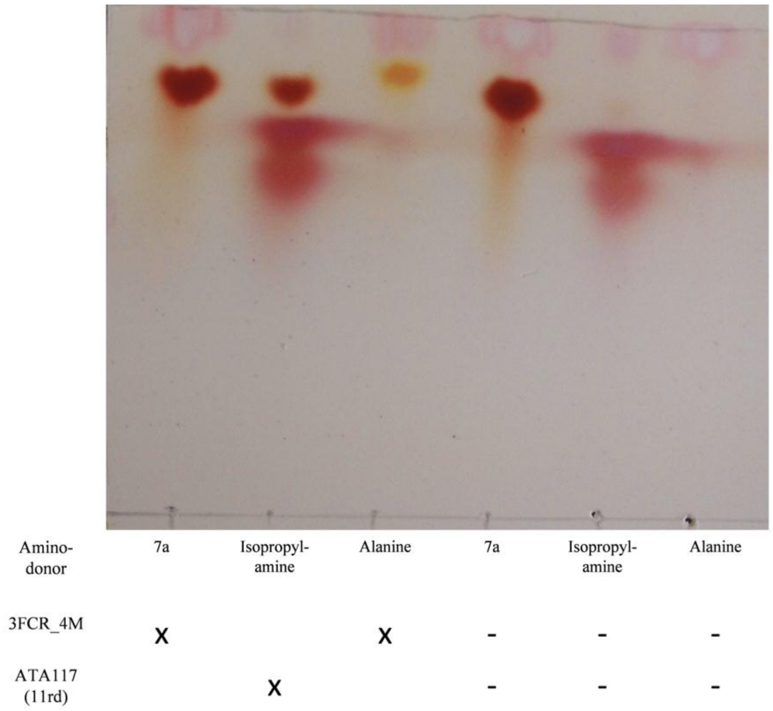

Figure S2 Pre-test of different amino donors **7a**, isopropylamine and alanine with **3** as amino acceptor. Reaction was performed at 30 ℃ in 50 mM HEPES buffer pH 7.5. 3FCR_4M as well as ATA117 11Rd were tested (**-** without enzyme). Concentration of the different amino donors: **7a** 50 mM, isopropylamine 400 mM and alanine 250 mM. The concentration of the substrate **3** was 10 mM. In yellow-orange the product **2** and in red-orange **7a** spots can be seen. The samples were taken after 24 h. ATA117 11Rd forms the unexpected product **7a** with isopropylamine as amino donor.

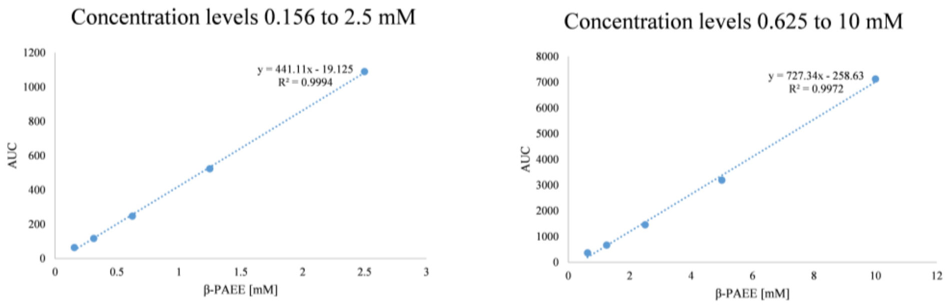

Figure S3 Gas chromatography analysis for the detection of β-phenylalanine ethyl ester. Solutions of different concentrations were prepared in buffer (pH 7.5) and extraction was performed using EtOAc. The extracted **2** in the organic phase (EtOAc) was analyzed using GC. The resulting peak area (AUC) at 28.9 min, detected by FID, was utilized for linear calibration.

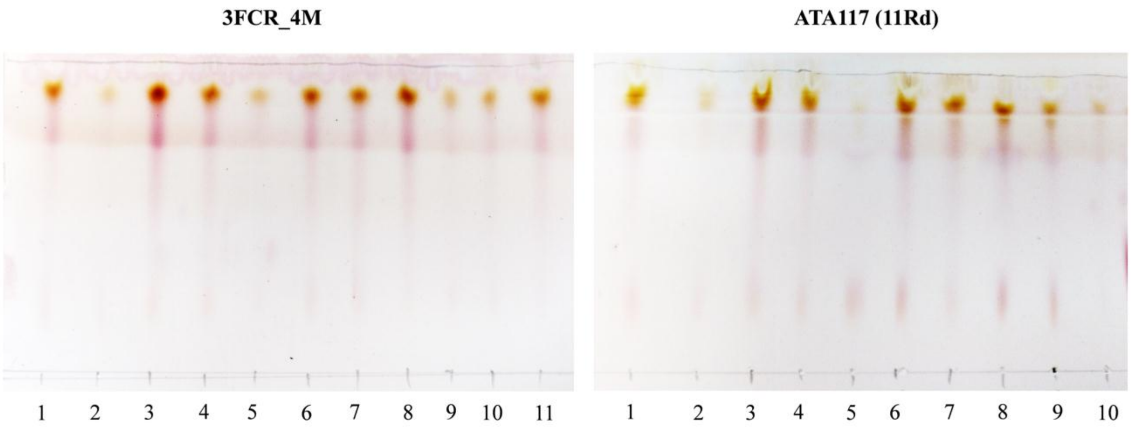

Figure S4 Design of Experiments varying DMSO, *o*-xylylenediamine and β-keto ester concentrations. The experiments were performed at 30 ℃ in 200 µL scale. The brown-yellow spot indicates the formation of **2**. The results were confirmed using GC. The concentrations of the mixture ingredients are reported in Table S2 and S3.

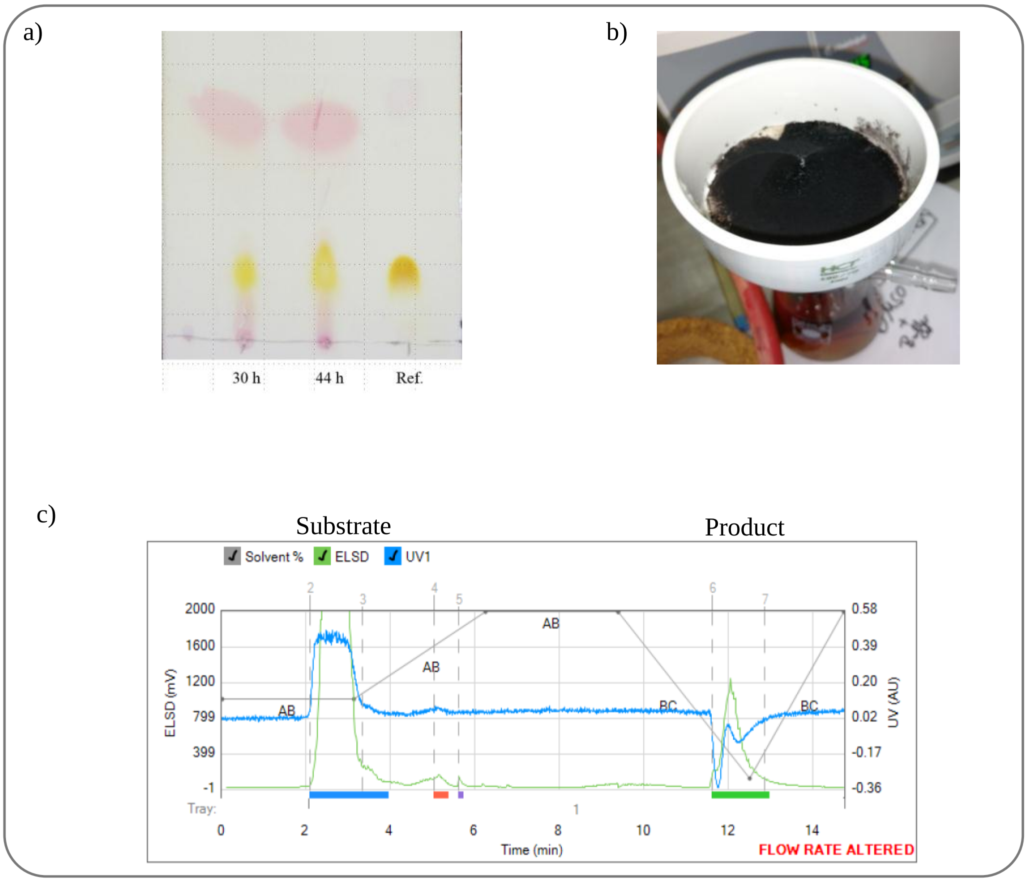

Figure S5 Reaction batch on a 200 mL scale after 48 h of reaction time using 3FCR_4M. a) Samples were taken during the whole reaction time (yellow spot indicates product). b) A strong discoloration was observed. c) Chromatogram of the flash chromatography purification using solvent gradient. Product was detected by ELSD signal

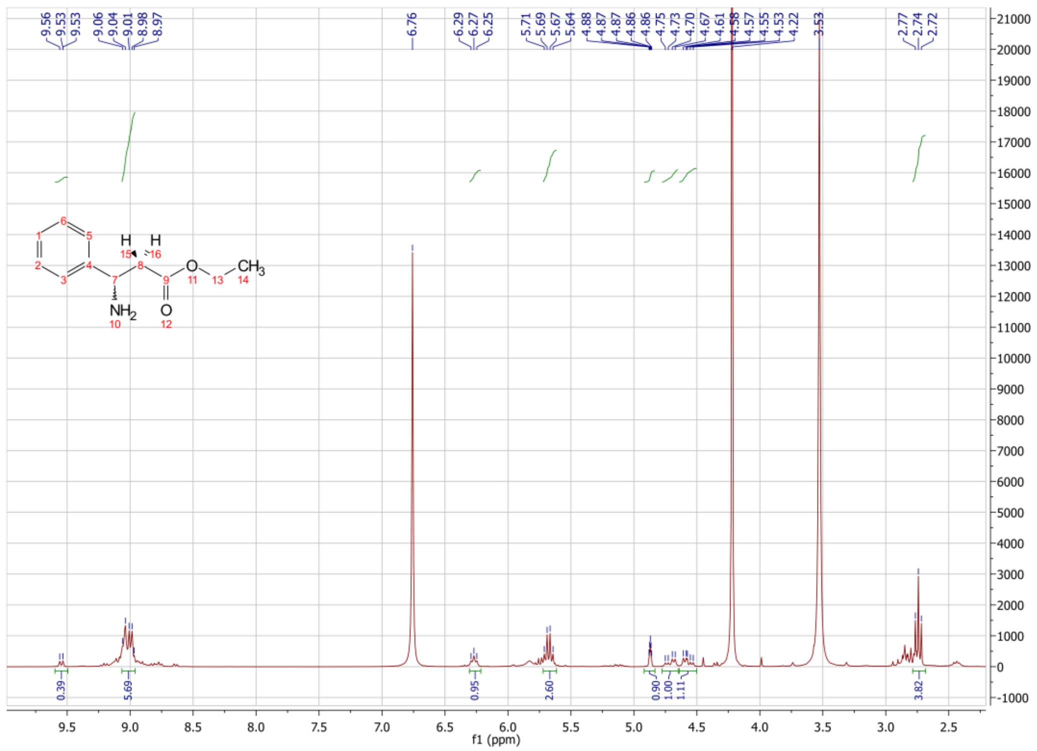

Figure S6 ^{1}H-NMR-spectrum of the product after purification using flash chromatography.

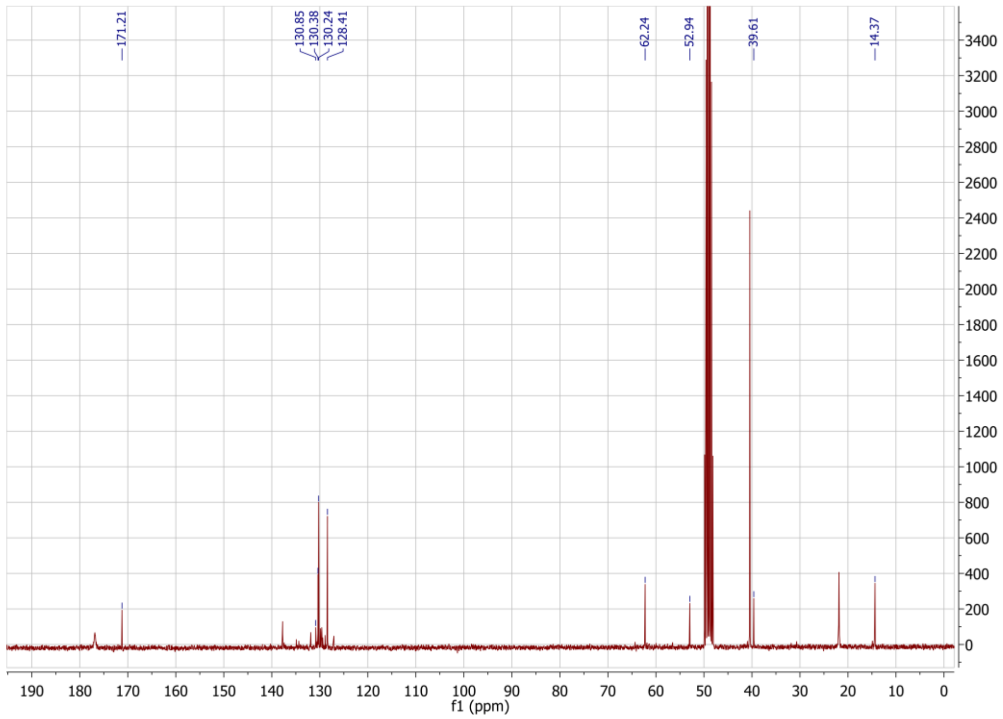

Figure S7 ^{13}C-NMR-spectrum of the product after purification using flash chromatography

Table S1 ω-TA plasmids and host organism of the transaminases for the β-phenylalanine ethyl ester screening. Expressions of the different ω-TA variants were performed in 96-deepwell plates using the expression host *E. coli* BL21 (DE3) and the indicated antibiotics and inductors.

**Source**	**Abbr.**	**Vector**	**Resistance**	**Inducer**	**Fold type***
*Ruegeria pomeroy*i	3HMU	pET22b	Amp	IPTG	I
Ruegeria sp. TM1040	3FCR	pET22b	Amp	IPTG	I
Mesorhizobium loti	3GJU	pET22b	Amp	IPTG	I
Rhodobacter sphaeroides KD131	3I5T	pET22b	Amp	IPTG	I
Vibrio fluvialis	VibFlu	pET24b	Kan	IPTG	I
Mesorhizobium sp. LUK	2YKY	pET28b	Kan	IPTG	I
Chromobacterium violaceum	Cvi	pET28a	Kan	IPTG	I
Aspergillus oryzae	AspOry	pGASTON	Amp	Rhamnose	IV
Aspergillus terreus	AspTer	pGASTON	Amp	Rhamnose	IV
Mycobacterium vanbaalenii	MycVan	pGASTON	Amp	Rhamnose	IV
Penicillium chrysogenum	PenChr	pGASTON	Amp	Rhamnose	IV
Burkholderia sp.	BurSp	pGASTON	Amp	Rhamnose	IV
Rhizobium etli	RhiEtl	pGASTON	Amp	Rhamnose	IV
Hyphomonas neptumium	HypNep	pGASTON	Amp	Rhamnose	IV
Gamma proteobacterium	GamPro	pGASTON	Amp	Rhamnose	IV
Labrenzia alexandrii	LabAle	pGASTON	Amp	Rhamnose	IV
Marimonas sp.	MarSp	pGASTON	Amp	Rhamnose	IV
Nocardia farcinica	NocFar	pGASTON	Amp	Rhamnose	IV
Rhodoferax ferrireducens	RhoFer	pGASTON	Amp	Rhamnose	IV

Bacillus sp.	D-AlaTA	pGASTON	Amp	Rhamnose	IV
Aspergillus fumigatus	AspFum	pET22b	Amp	IPTG	IV
Gibberella zeae	GibZea	pET22b	Amp	IPTG	IV
Neosartorya fischeri	NeoFis	pET22b	Amp	IPTG	IV
Rhodobacter sphaeroides	RhoSph	pET22b	Amp	IPTG	IV
Jannaschia sp.	JanSp	pET22b	Amp	IPTG	IV
Mesorhizobium loti	MesLoti	pET22b	Amp	IPTG	IV
Roseobacter sp.	RosSp	pET22b	Amp	IPTG	IV
Arthrobacter sp. KNK168	ATA117	pET22b	Amp	IPTG	IV
Arthrobacter sp. KNK168 (mut)	ATA117 11rd	pET22b	Amp	IPTG	IV

* Fold type of PLP-dependent enzymes (for ω-TA relevant only I and IV)

Table S2 Design of Experiments for the optimization of β-phenylalanine ethyl ester production using 3FCR_4M. The concentrations of *o*-xylylenediamine (**4**), β-keto ester (**3**) and DMSO were varied. The reactions were conducted at 30 ℃ for 24 h. The concentration of β-phenylalanine ethyl ester was determined using GC.

RUN	4 [mM]	3 [mM]	DMSO [%v/v]	Conversion
**1**	100	40	10	6%
**2**	10	40	10	6%
**3**	100	40	30	6%
**4**	55	25	20	11%
**5**	10	10	10	27%
**6**	55	25	20	11%
**7**	100	10	10	23%
**8**	100	10	30	15%
**9**	10	40	30	5%
**10**	10	10	30	18%
**11**	55	25	20	12%

Table S3 Design of Experiments for the optimization of β-phenylalanine ethyl ester production using ATA117 11Rd. The concentrations of *o*-xylylenediamine (**4**), β-keto ester (**3**) and DMSO were varied. The reactions were conducted at 30 ℃ for 24 h. The concentration of β-phenylalanine ethyl ester was determined using GC.

RUN	4 [mM]	3 [mM]	DMSO [%v/v]	Conversion
**1**	100	40	10	1.6%
**2**	10	40	10	1.5%
**3**	100	40	30	1.8%
**4**	55	25	20	3.2%
**5**	10	10	10	6.5%
**6**	55	25	20	3%
**7**	100	10	10	6.8%
**8**	100	10	30	5.5%
**9**	10	40	30	1.4%
**10**	10	10	30	3.1%
**11**	55	25	20	2.5%
